# The Role of Photodynamic Therapy in Triggering Cell Death and Facilitating Antitumor Immunology

**DOI:** 10.3389/fonc.2022.863107

**Published:** 2022-05-27

**Authors:** Liuchang Tan, Xiaoxiao Shen, Zhiqiang He, Yuangang Lu

**Affiliations:** Department of Plastic Surgery, Daping Hospital, Army Medical University, Chongqing, China

**Keywords:** photodynamic therapy, immunotherapy, cell death, tumor, immune response

## Abstract

Cancer is a major threat to human health because of its high mortality, easy recurrence, strong invasion, and metastasis. Photodynamic therapy (PDT) is a promising minimally invasive treatment for tumor. Compared with traditional treatment methods, PDT is less invasive and does not easily damage normal tissues. Most of the effects of this treatment are due to the direct effects of singlet oxygen together with reactive oxygen species. PDT can provide the source of active oxygen for the Fenton reaction, which enhances ferroptosis and also improves the efficacy of PDT in antitumor therapy. Additionally, in contrast to chemotherapy and radiotherapy, PDT has the effect of stimulating the immune response, which can effectively induce immunogenic cell death (ICD) and stimulate immunity. PDT is an ideal minimally invasive treatment method for tumors. In this paper, according to the characteristics of anti-tumor immunity of PDT, some tumor treatment strategies of PDT combined with anti-tumor immunotherapy are reviewed.

## Introduction

Cancer is a major threat to human health because of its high mortality, easy recurrence, strong invasion, and metastasis (1). Immunotherapy uses the body’s autoimmunity to remove and kill tumor cells, which has the characteristics of high selectivity and low adversarial reactions (2). At present, immunotherapy primarily includes three categories: cancer vaccine, adoptive T-cell therapy, and immune checkpoint blocking immunotherapy (3). Photodynamic therapy (PDT), as one of the most effective treatment modalities to tumor ablation, has attracted much attention due to its unique immunogenicity.

Three essential contents are included in PDT procedures: non-toxic photosensitizers (PSs), tissue oxygen, and harmless light (4). The absorption properties of PSs are matched to specific wavelengths of light (5). PDT is a two-stage procedure, which initially uses a PS and then uses local directional light, with the aim of confined tumor destruction. Usually, these PSs are composites with macrocyclic configurations, which contain chlorins, porphyrins, and other tetrapyrroles. When PSs, excited by the light of specific wavelengths of laser, react with molecular oxygen, activated PSs transfer energy to the neighboring molecular oxygen, culminating in the generation of the efficient singlet oxygen. Those unstable singlet oxygens are capable of directly causing cellular damage to organelle membranes and cell membranes where they are produced and can damage targeted tumor cells and vasculatures *via* apoptosis, necrosis, and facilitated antitumor immunity (6, 7). Compared with normal tissues, this selectivity is achieved by the accumulation of PSs in tumors and the fact that light is limited to a specific location. The tumor microenvironment can be fundamentally separated into (1) the main part of the solid tumor composed of tumor cells; (2) vasculature around the tumor construction stamped by endothelial cells; and (3) interstitial space composed of immune cells as well as stromal cells (8). There are three mechanisms of PDT-mediated tumor destruction *in vivo*: (1) direct killing of tumor cells, (2) damage to the vasculature, and (3) inflammation and immune responses (9).

Recent studies find an important role of immune response in PDT, which attracted growing interest and inspired studies of induction and promotion of the antitumor immune response combined with PDT.There are three components in the review: mechanism and effect about antitumor immunity combine PDT, recent development of applying antitumor immunity to get better therapeutic results, and opportunities and challenges to facilitate PDT antitumor immunotherapy.

## Mechanism of PDT-Induced Tumor Injury

**Figure 1:**
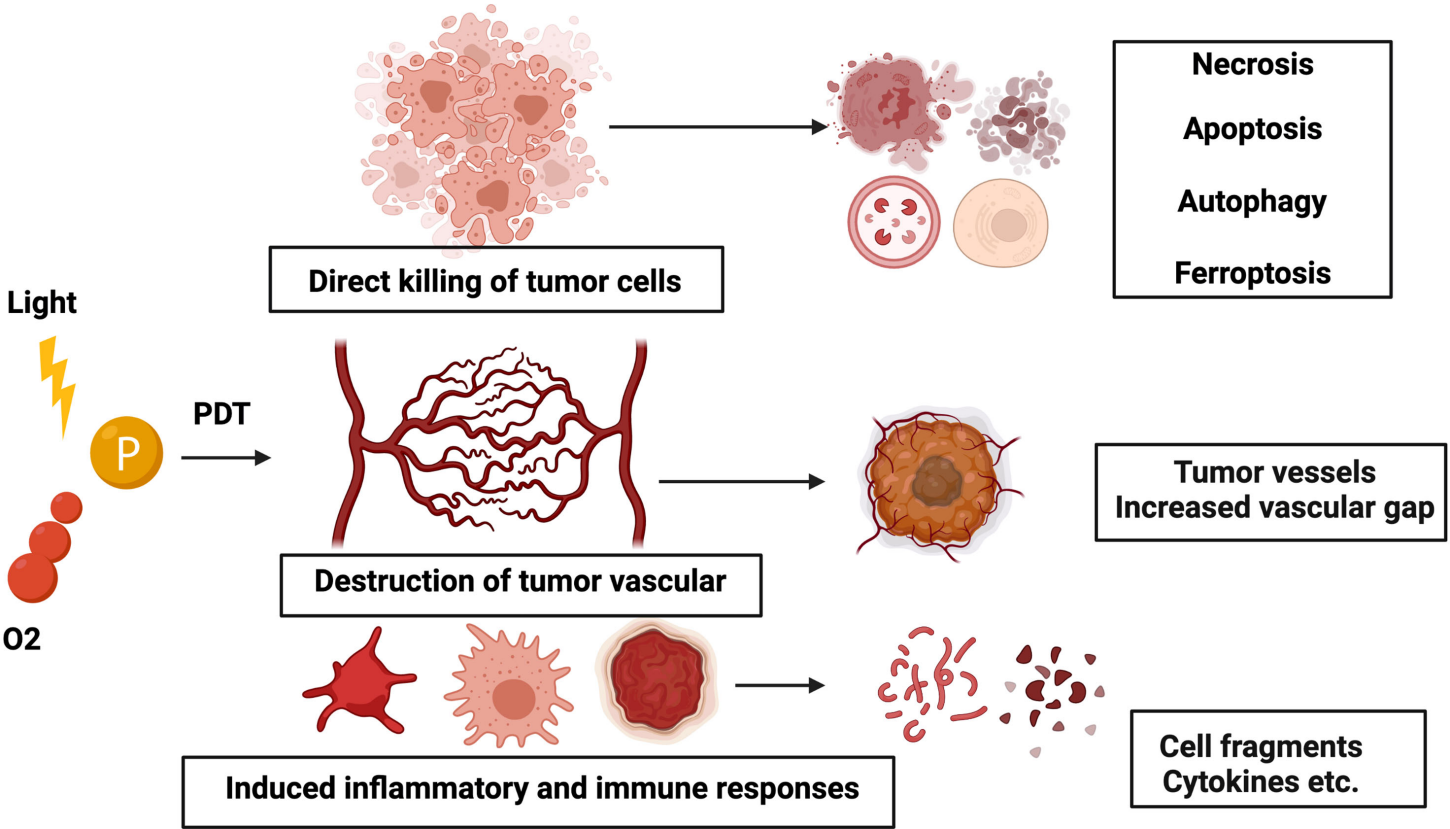
Schematic illustration of the mechanism of PDT. Three mechanisms of PDT-mediated tumor injury *in vivo*: (1) direct killing of tumor cells, (2) damage to the vasculature, and (3) inflammation and immune responses.

At first, research finds that PDT-induced injury to tumors was restricted to the treatment area. When PSs, the selective intake by tumor cells, are exposed to light, the generated reactive oxygen species destroy tumors using three mechanisms: causing irreversible direct injury to the tumor cells, PDT-mediated injury towards endothelial cells causing damage to the tumor microvasculature and capillaries, and triggering an acute inflammation and immune response (10) (**Figure 1**).

### (1) Direct Killing of Tumor Cells

Reactive oxygen species induced by PDT could oxidate the sub-cellular organelles and injure the microvasculature, causing light-induced cell death. Among them, PDT-induced killing of tumor cells involves necrosis, apoptosis, ferroptosis, etc. Mild doses of PDT in the mitochondria could trigger apoptosis, while necrosis is induced when excessive doses of PDT are administered in the plasma membrane area. Apoptosis was introduced *via* various pathways following PDT-induced destruction of organelles, which is a controlled mechanism of cell death, and its process is highly regulated (11, 12). Photodamage-induced mitochondrial membrane permeability leads to cytochrome c leakage into the cytoplasm, which, in turn, activates caspase (an extremely conserved family of cysteine-dependent aspartate-specific proteases)-mediated apoptosis pathway (13). Apoptosis is a controlled mechanism of cell death, and its process is highly regulated. Opposite to the apoptotic pathway, necrosis is studied through uncontrolled cell death modality, which occurs by applying high doses of chemical substances and/or physical stress. In PDT, the activation of most necrosis pathways is interceded by high-concentration irradiation or high-dose PS. Photodamage to the plasma membrane will lead to the leakage of intracellular substances into the direct environment, leading to inflammation (14).

The double-membrane structure of the autophagosome engulfs the damaged granules, and the autophagosome fuses with the lysosomes to break down its components (15). Even though autophagy attempts to repair the damage to organelles and cells caused by low-dose PDT, it may also lead to cell death when its protective ability either is suppressed or malfunctions due to, for instance, lysosomal damage. Ferroptosis is characterized by the iron-dependent accumulation of lipid peroxides by obscuring the system xc-cystine/glutamate antiporter or inhibiting glutathione peroxidase 4 (GPX4). Oxidative modulation of cellular membrane phospholipids increases membrane permeability, which will carry plasma membrane rupture and finally cell death. In the type I process, the excited PS transforms into a triplet state and combines with oxygen to form hydroxyl radicals and superoxide. In the type II process, the excited PS transforms into singlet oxygen. PDT can serve as the source of H_2_O_2_ for the Fenton reaction and can serve as a source of lipid ROS additionally (16).

PDT can activate a variety of cell death reactions and avoid the dilemma of anti-apoptotic cells in tumors, which is the main difficulty in the treatment of other cancers (12). Elucidation of the in-depth role of PS and its related reaction on the molecular level is essential to understanding PDT.

### (2) Damage to the Vasculature

Neovascularization is the key process of tumorigenesis and development. The damage to existing blood vessels or the inhibition of neovascularization has adverse effects on tumor proliferation (17). In the early stage of PDT, events such as platelet aggregation, edema formation, thrombus formation, thromboxane release, and the initiation of the complement cascade will directly damage endothelial cells, followed by vessel contraction, resulting in the exposure of the basement membrane and vascular leakage (18). In PDT-treated tumors, the initiation of complement cascade and the vascular endothelium’s production of membrane attack complex (MAC) may be the basis of blood supply failure (19). Meanwhile, PDT blocks endothelial cells from releasing nitric oxide (NO) and promotes vasoconstriction further, which will lead to ischemia-related cell death.

First of all, tumor cells cannot synchronize with anti-angiogenic and pro-angiogenic factors, which leads to an unusual vascular structure, enlarged blood flow disorder, and vascular space. At the same time, due to the mechanical characteristics of the PS, it shows good affinity with vascular endothelial cells and tumor endothelial cells. This oxidative stress promotes complement activation and infiltration of neutrophils and other inflammatory cells in previously ischemic regions. Vascular endothelial injury leads to hypoxia. The increased hypoxia-inducible factor-1a (HIF-1a) promotes immune escape and immunosuppression by activating tumor-associated macrophages (TAMs), myeloid-derived suppressor cells (MDSCs), lymphocytes, and dendritic cells (DCs).

### (3) Inflammation and Immune Responses

PDT of tumors produces a variety of inflammatory mediators and molecules, called damage-associated molecular patterns (DAMPs), to signal the immune system, analogous to pathogen-associated molecular patterns (PAMPs). PDT also triggers a variety of cell-signaling cascades, cytokines, cell fragments, and inflammatory mediators. PDT also accelerates the interaction between the innate arm and the adaptive arm of the immune system (14, 20). The process changes the tumor microenvironment by stimulating the expression of acute-phase response mediators and pro-inflammatory in irradiated space. This cascade process activates general inflammation, innate immunity, and, in turn, adaptive immunity.

PDT, as a limited treatment, destroyed the tumor structure directly and also initiated an acute inflammatory response indirectly. The oxidative stress induced by PDT can increase the delivery of heat shock proteins (HSPs) and the release of inflammatory transcription factors and inflammatory cytokines (14). Leukocytes infiltrate the tumor site and produce proinflammatory factors and cytokines. The PDT mechanism, which promotes a strong inflammatory response, induces neutrophils to flow into the treatment site rapidly, thus improving the tumor response rate and enhancing immunity. Neutrophils directly act on photodamaged cells and then remove photodamaged tumor cells, while affecting the survival/proliferation of tumor-specific T cells and mediating the creation of anti-tumor immunity after PDT (21). Sluiter et al. noticed that PDT neutrophils adhere and aggregate to the microvascular wall *in vivo*, which provides necessary information for the correlation of neutrophils in anti-tumor response (22). The activation of the complement system has become a mediator in anti-tumor treatment, which also increases the secondary inflammatory mediators, such as cytokines IL-1β, IL-6, IL-10, TNF-α, and G-CSF, leukotrienes, thromboxane, prostaglandins, histamine, and coagulation factors (23, 24). The complement activation forms transmembrane channels, destroys the completeness of plasma membrane, and leads to cell death and lysis.

PDT can stimulate the innate arm and adaptive arm of the immune system because the exposure of tumor cells is essentially related to immune cells (25). The innate immune response system includes the complement cascade system, cellular elements, phagocytes (macrophages, neutrophils, and DCs), and natural killer cells. The PDT-related activation of the innate immune system combines with recruitment, cytokine release, activation of innate immune cells, and complement activation (26).

## PDT-Induced Antitumor Immunity on Different Immune Cells

As phagocytic cells, macrophages can directly kill tumor cells, which have the ability to differentiate into M1 and M2, two macrophage phenotypes. Recruitment and activation of macrophages enhanced by PDT potentiate immunity, and while M1 macrophages are mainly concerned with immune initiation and invasion, M2 macrophages are involved in promoting tumor development and wound curing. M2 macrophages, known as TAMs, are essentially an effective target for anti-tumor treatment. It was found that photodynamically killed tumor cells could indirectly stimulate the antitumor activity of macrophages (27). Moreover, macrophages produce chemokines, cytokines, and other essential mediators in the procedure of PDT-mediated inflammation.

DCs, known as the most effective antigen-presenting cells (APCs), are the key factor of the immune response, which link the specific and non-specific immune reaction. The activation and the phenotypic and functional maturation of DCs are crucial for tumor-associated antigen (TAA) expression and cytotoxic T lymphocyte (CTL) formation (28). In the anti-tumor immunity, TAAs are captured by DCs through a variety of pathways, including uptake of apoptotic tumor cells, necrotic tumor cell fragments, or release of soluble tumor antigens, especially those associated with HSPs (29). At the cell surface, mature DCs deliver peptide-major histocompatibility complex (MHC) proteins as well as appropriate costimulatory molecules, which mainly enables CD4^+^ T helper cells and CD8^+^ to proliferate, activate, and differentiate into effector cells, and initiates an adaptive immune response (30). Jalili et al. found that co-culture of DCs with PDT-treated cells can lead to active phagocytosis, which can support the maturation of DCs, the expression of costimulatory molecules, and the release of proinflammatory cytokines (31). PDT could build a valuable environment for antigen loading and DC activation at the tumor site.

**Figure 2:**
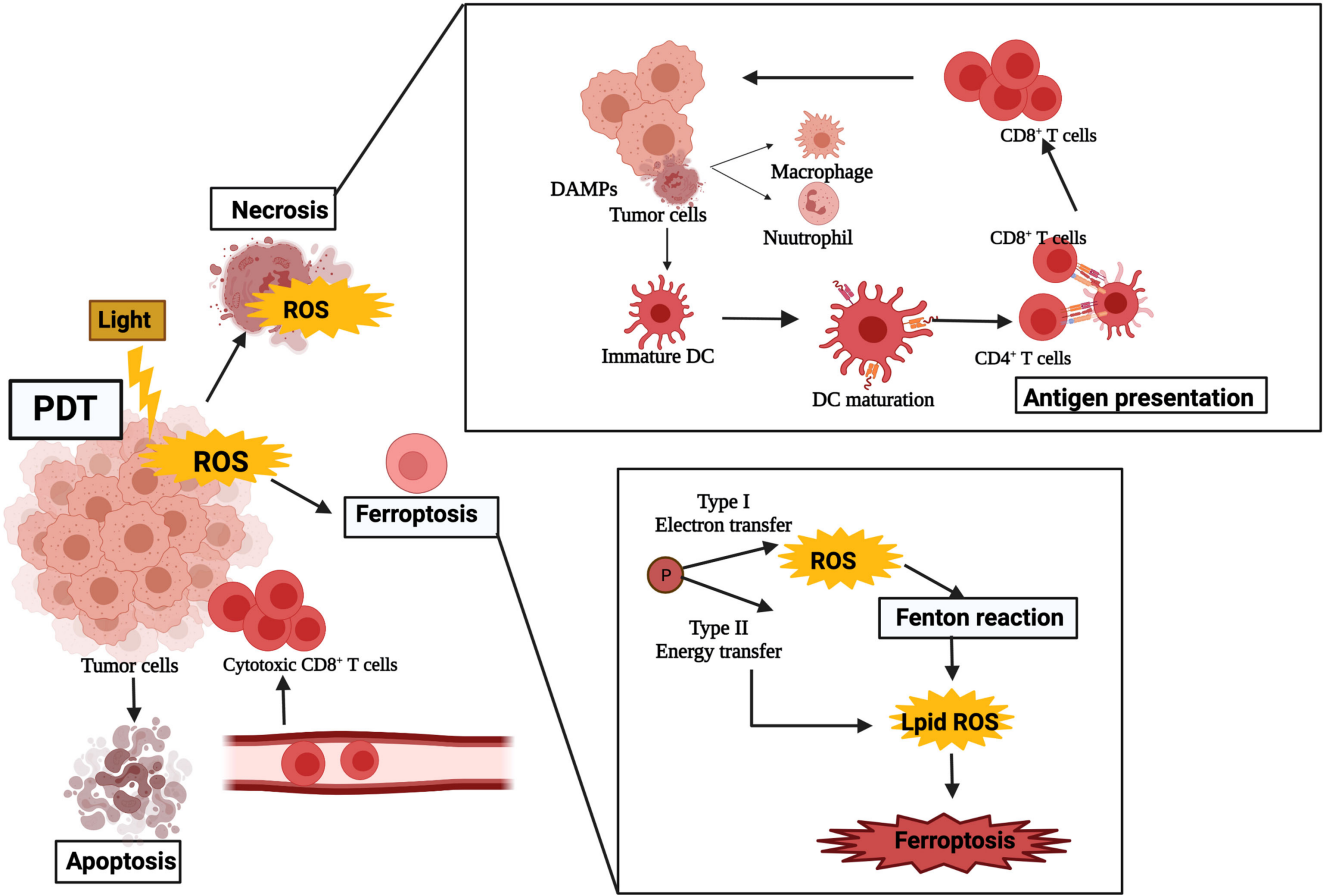
The stimulation of photodynamic therapy on the immune system after treatment. The ROS can cause damage to tumor cells and lead to the release of DAMPs, which, in turn, activate host immune cells such as neutrophils, dendritic cells, and macrophages. Immature dendritic cells infiltrating peripheral tissues engulfed apoptotic tumor cells and oxidatively damaged cells. DAMP, damage-associated molecular pattern; DC, dendritic cell; ROS, reactive oxygen species.

Activation of T lymphocytes plays a significant role in killing tumor cells in the process of tumor immunotherapy, especially cytotoxic CD8^+^ T effector cells presented by MHC class I molecules. The adaptive arm of the immune system includes APCs and antigen and is triggered by recognizing endogenous and exogenous antigens. APCs can activate naive T cells to convert cytotoxic tumor-specific T lymphocytes (CTLs) and antigen recognized by B cells for antibody production. PDT-induced immunogenic apoptosis of tumors cells through triggering of DCs results in the activation of specific T-cell reactions. DCs are the most effective APCs to link innate immune responses and adaptive immune responses. DCs can recognize antigens released by apoptotic tumor cells and travel to lymph nodes, where the TAAs were presented to immature T cells, which turned into CTLs, cleared residual tumor cells, and activated adaptive immunity. Mature DCs stimulate peptide-MHC proteins, mainly activate CD4^+^ T helper cells and CD8^+^ to CTLs, and introduce an adaptive immune response (32). They trigger naive T cells to become tumor-specific cytotoxic T cells that can destroy the tumor in return (**Figure 2**). In the early stage of tumor ablation, although specific immune responses do not seem to be principally effective, they may provide long-term control of tumor cells. The optimal anti-tumor therapy involving PDT will include immunotherapy methods that will accelerate the progress of concomitant immunity.

## PDT and Immune Response Against Cancer

PDT can induce the immunogenic death of tumor cells [immunogenic cell death (ICD)], stimulate the release of tumor-related antigens in tumor cell residues, and further spread the activation and infiltration of antigen-specific T cells, as well as their proliferation. It is one of the most widely studied minimally invasive anti-tumor treatment methods, which is usually combined with immunotherapy to improve the efficiency of its anti-tumor treatment. Most studies suggest that PDT-induced anti-tumor immunity is due to the release of tumor antigen and DAMPs after PDT induced oxidative stress in the tumor site. The reported samples of DAMPs released after PDT are HSPs (such as HSP60, HSP70, HSP90), adenosine-triphosphate (ATP), high mobility group box 1 (HMGB1), and calreticulin (CRT) (33), which are translocated and upregulated to the membrane, and which, as a danger signal, can be acknowledged and neutralized by innate immune cells, and then trigger the innate immune response (34, 35). One of the DAMP molecules, CRT, enhanced the tumor response of immunocompetent mice rather than immunodeficient mice after PDT administration (36). CRT was found to bind PDT-damaged cells and stimulate migration and phagocytosis by DCs and macrophages. DAMPs activate host immune cells such as macrophages, neutrophils, and DCs. PDT-induced expression of DAMPs may be a powerful way of killing tumor cells through immune reaction.

### (1) PDT and Immune Checkpoint Blockade Therapy

Tumor growth and development are often accompanied by a tumor immunosuppressive microenvironment. This immunosuppressive network includes regulatory T cells (Tregs), MDSCs, and type 2 (M2) macrophages, as well as immunosuppressive cytokines (37). Immune checkpoint molecules are considered major anticancer immunotherapy targets because of their negative immunomodulatory effects. Immune checkpoint blockade (ICB) therapy is one of the effective methods to reverse the tumor immunosuppressive microenvironment. It can block the invasion of cancer cells by inhibiting Tregs and activating CTLs (38, 39).

Some studies suggest that PDT can induce immunogenicity and enhance tumor T-cell infiltration, which is an effective immunogenic therapy. It can make tumor sensitive to ICB treatment and improve tumor treatment efficiency (40). However, ICB treatment still needs to face challenging conditions such as a low response ratio and immune-related adverse events (irAEs). Improving immunogenicity and tumor T-cell infiltration is one of the key objectives of ICB therapy (41). Duan et al. also used monoclonal antibody programmed death-1 (PD-1) to block the inhibitory receptors of tumor cells, which escape the host’s immune response through T lymphocyte exhaustion (42). Therefore, the cooperative effect of immune checkpoint inhibition and PDT could promote the suppression of primary tumors and the elimination of metastatic lesions (43). The regulation of gene expression through epigenetic reversion is also helpful to enhance the anti-tumor effect of PDT. The basic components of immune response, such as TAA or MHC I, are usually downregulated in tumors. The expression of MHC I and P1A can be restored by altering DNA methylation and improving consequential immune cell recognition and tumor antigen presentation. The results increased expression of P1A, and enhanced adaptive immune response after PDT, anti-tumor immune-mediated reactivation rejection, and long-term survival.

### (2) PDT and Immunostimulators

Immunostimulators are a class of substances that can promote the body’s immunity and play a role in enhancing the photoimmunotherapy of tumors, which are often used as adjuvants to enhance cancer vaccines (44). In order to elicit an immune response, one mode is to use PDT in combination with immunostimulators.

**Figure 3:**
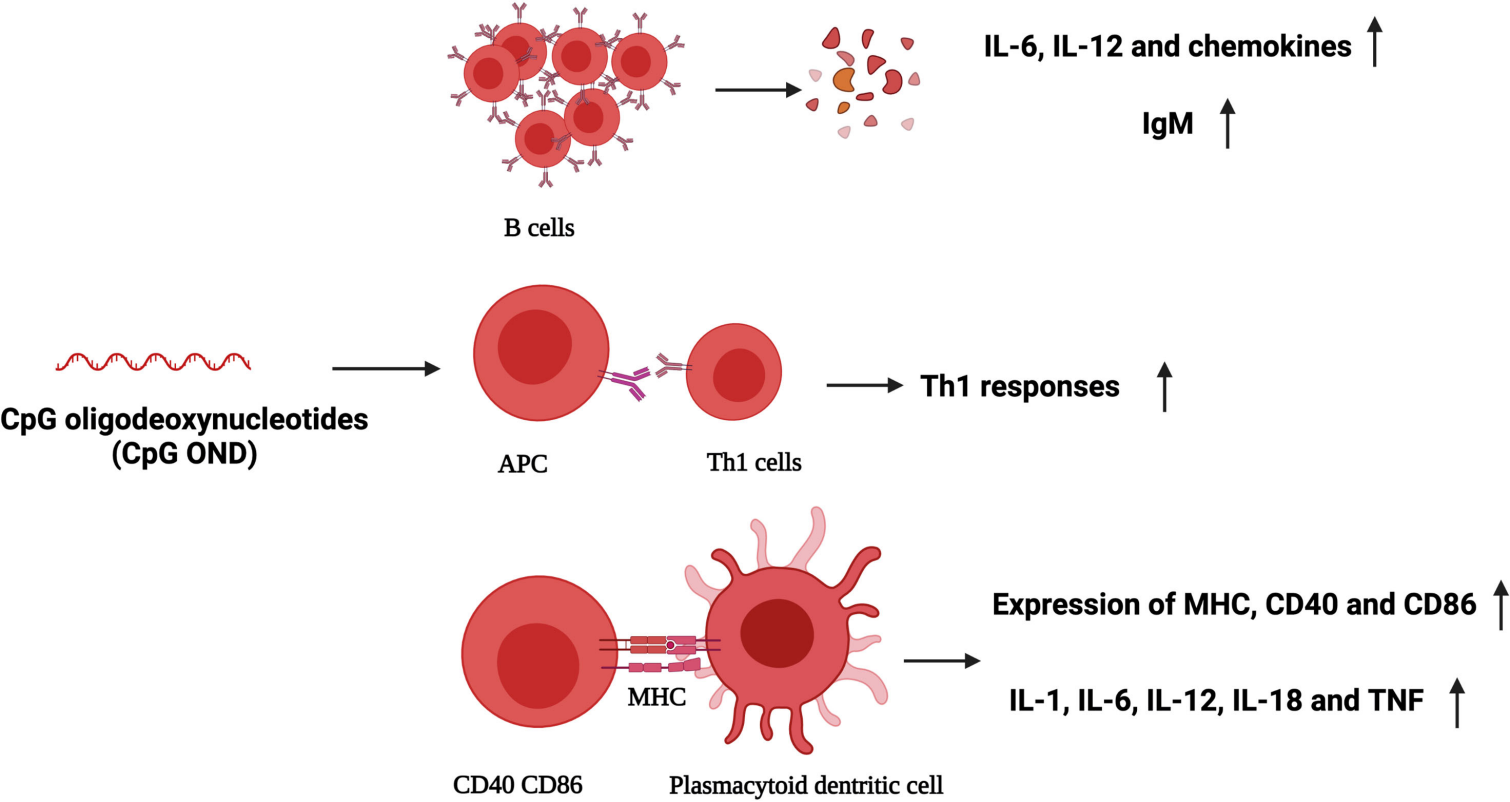
CpG oligodeoxynucleotides (ODNs) activate plasmacytoid dendritic cells and human B cells directly, and stimulate the creation of T helper 1 (TH1)-type cytokines. IL, interleukin; IgM, immunoglobulin M; TH1, T helper 1 (TH1)-type cytokines; CD40, CD86, markers of activation and maturation of dendritic cells; MHC, major histocompatibility complex; TNF, tumor-necrosis factor.

Effective immunostimulants in combination with PDT showed an excellent antitumor effect, such as cytokines in inflammatory cascades, namely, hormones, growth factors, complement activators, microbial vaccines, and other exogenous immunoadjuvants such as BCG, glycated chitosan, TLR-2/4, TLR-7, CpG oligodeoxynucleotide, and resiquimod. The tumor antigen released after PDT and the co-existence of these immunostimulants can significantly enhance the anti-tumor immune response and improve the therapeutic effect. CpG oligodeoxynucleotide is a toll-like receptor (TLR) agonist with a strong immunostimulatory activity. It can bind to TLR9 and promote the expression of costimulatory molecules and the secretion of inflammatory cytokines, such as B cells, natural killer cells, monocytes, and macrophages (45). Meanwhile, peritumoral injection of CpG oligodeoxynucleotides can lead to the migration of primary DCs towards the tumor, showing improved maturation, phagocytosis, and antigen presentation to T cells. In addition, CpG ODNs can directly inhibit the immunosuppressive function of MDSCs and make them differentiate into macrophages with anti-tumor activity (46). Some researchers have constructed a CpG nanoscale metal framework that can effectively adsorb anions to cure breast cancer founded on the high immunogenicity of PDT. In order to verify the therapeutic effect of CpG ODNs combined with PDT, CpG ODNs were co-loaded on the nanoscale metal framework to transfer CpG and promote DC maturation. While CpG primes immature DCs to activation and maturation, researchers described that peritumoral injected CpG enhanced local tumor control and displayed a survival benefit in mice with PDT (47). These results show that the effective delivery of PDT and CpG can activate the anti-tumor immunity (**Figure 3**). The combination of PDT and CpG has a synergistic anti-tumor effect and can effectively inhibit the growth of tumor, and the growth rate is reduced, leading to a higher survival ratio.

The US Food and Drug Administration has permitted resiquimod (R848) as an immunomodulator for skin cancers and pathologies in clinical trials. Resiquimod (R848) is an aquaphobic imidazoquinoline-like molecule that can bind to TLR7 and TLR8. The motivation of TLR7/TLR8 by resiquimod may activate APCs, upregulating the co-stimulatory factors CD86 and CD80, which are the trademarks of DCs in maturation, altering the phenotype from immunosuppressive to immunogenic (**Table 1**) (48, 49). Mature DCs then secrete numerous pro-inflammatory cytokines, including tumor necrosis factor-α (TNF-α) and interleukin-6 (IL-6), controlling anti-tumor immune responses. In conclusion, the PDT adjuvant of immune adjuvant has the ability to inhibit tumor metastasis and recurrence, and has broad research potential.

**Table 1:** Delivery routes (intratumoral injection and intravenous injection) for PDT and immunostimulants.

	Intratumoral injection	Intravenous injection
Immunostimulants	Glycated chitosan	Photosensitizers
	Mycobacterial cell wall extract GM-CSF Schizophyllan	GC-MAF
	CpG-ODN Zymosan Gamma inulin OK-432 Corynebacterium parvum Bacillus Calmette-Guerin	Urokinase Streptokinase Cyclophosphamide
	G-CSF	

GM-CSF, granulocyte macrophage colony stimulating factor; CpG-ODN, CpG containing oligodeoxynucleotide; OK-432, killed Streptococcus pyogenes; G-CSF, granulocyte colony stimulating factor; GC-MAF, macrophage activating factor. Imiquimod is applied topically.

### (3) PDT and DC Effective Vaccine

In anti-tumor immunotherapy, antigen cross-expression and strong antigen-specific CD8+ T-cell immune response are the key factors affecting therapeutic efficiency. In order to enhance the anti-tumor immune response in the procedure of PDT, in addition to the classic combined application strategy mentioned above, there are many immune enhancement methods, such as antigen capture, enhanced antigen expression, enhanced antigen presentation, and immune vaccine. The relevant method to utilize the immune stimulation effect of PDT is the PDT-generated cancer vaccines (50).

This strategy of transforming immature DCs into operative vaccine adjuvants throughout PDT reaction is enough to introduce adaptive T-cell response *in vivo* (51). Cell lysates produced by PDT are more effective in inducing immune responses than lysates produced by ultraviolet or ionizing radiation or freeze–thaw cycles (52). Among them, the cell lysate produced by PDT can activate DCs and induce a strong cytotoxic T-cell response, which is tumor specific and has a greater potential to become a cancer vaccine. Unlike traditional vaccines, which directly introduce microorganisms to produce protective antibodies that can be recognized by the human body, cancer vaccines expose tumor cells to lethal doses of radiation and then introduce these killed tumor cells into animals (53, 54). Instead of the standard PDT for solid tumors, tumor tissues are treated *in vitro* by PDT to produce vaccine materials, which are injected into patients with tumors. It is expected that the host’s immune system can recognize the killed tumor cells and produce immunity.

An alternative to using PDT-treated tumor cells or tumor cell lysates as vaccines is to use live DCs. By exposing DCs to PDT-treated tumor cells or *in vitro* lysates, they can phagocytose cells or free antigens and display them. Compared with the injection of whole tumor cell lysates that have almost no immune effect, the injection of PDT-induced cell lysates *in vitro* stimulated DCs and can generate specific anti-tumor immunity against fully established solid breast tumors in mice and prolong survival time (55). At present, the apoptotic tumor cells produced by PDT have been used as tumor antigen binding to DCs, and the tumor cells treated by PDT have been used as a DC vaccine to form a PDT-DC vaccine. This kind of PDT-DC vaccine can effectively eradicate tumor, trigger a stronger anti-tumor preventive effect, and enhance T lymphocyte response, which is an ideal combination therapy strategy (56). Some scholars have found that DCs initiated with specifically isolated MHC I binding antigen after PDT of isolated tumor cells are more effective in mediating immune responses (57). Filtering out non-specific and possible immunosuppressive antigens and cytokines from cell lysates left a more effective pool of anti-tumor antigens to present to DCs, which created a more tumor-specific vaccine compared to whole-cell lysate-primed DCs. More effective DC maturation, increased cytokine production, and improved antigen-specific CTL response *in vivo* were observed (57).

Zhang et al. used ovalbumin (OVA) as a model antigen and verified that the feasibility of ROS produced by laser irradiation can significantly enhance the cross-expression efficiency of OVA antigen (58). During PDT, ROS can not only stimulate the release of DAMPs as an immune adjuvant to promote the maturation of APCs, but also improve the efficiency of antigen cross-expression required by CD8^+^ T cells. In this study, bone marrow-derived dendritic cells (BMDCs) were sequestered from the tibia of mice and cultivated, which then were treated with antigen nanoparticles. The supernatant of b16-OVA cells was used to stimulate immature BMDCs. The results showed that compared with the control group, the expression of CD80 and MHC-II increased significantly in the antigen nanoparticle groups, which confirmed the function of ROS in promoting the maturing of BMDCs. Ni et al. have developed a type of nanoparticle co-loaded with Dox and Ce6 that can effectively induce ICD by using the cytotoxicity of Dox as well as the PDT material of Ce6, so as to better expose and spread TAA. The ROS produced in this procedure is expected to recruit DCs by simulating the inflammatory mechanism and form an *in situ* DC vaccine for the treatment of breast cancer (59). In addition to the effective recruitment of DC, the level of serum cytokines in mice increased, indicating that the *in situ* vaccine can effectively induce inflammatory response and trigger anti-tumor immunotherapy. Korbelika et al. used the SCCV II cells after PDT to prepare the vaccine and established the mouse SCCV II tumor model. The tumor growth of mice inoculated with the PDT vaccine significantly slowed down (60). At the same time, the level of MDSCs in spleen cells of mice was detected by flow cytometry. The outcomes showed that the PDT vaccine had an important effect on the amount of immunoregulatory cells in spleen. Although the PDT vaccine is still in its early stage, recent studies show that the PDT vaccine has the potential to become a useful adjuvant or main treatment for diverse cancers (61).

## Summary

PDT, modality by electron or energy transfer, is a non-invasive and effective method of tumor treatment that has broad application prospects. PDT is a successful intervention for the treatment of pre-malignant and early-stage diseases, and is considered an effective palliative treatment for advanced-stage diseases. Due to the limited light permeation and phototoxicity of PSs, monotherapy regimen for PDT did not achieve an expected therapeutic effect. PDT-mediated oxidative stress and some PSs may produce new and unique tumor antigens, which make cancer cells treated by PDT more immunogenic than those killed by other methods. PDT combined with immunostimulant approaches can significantly increase therapeutic efficacy. It should be pointed out that the combination of immune checkpoints and PDT may be a promising direction for future research. Recognizing the inherent potential of PDT and the mechanism of triggering host immune response, researchers studied the ways that various immunotherapy strategies are consistent with the improvement of PDT efficiency. However, it remains to be elucidated under what situations PDT-prompted immunity can be enhanced to overwhelm tumor immunosuppression and obtain enough anti-tumor response, and to what amount PDT-prompted anti-tumor immunity can result in thorough tumor rejection. In order to answer these questions, we need to know more about the pathways by which tumor cells escape host immune response. In the near future, PDT and immunotherapy will be more effective in clinical tumor treatment. Therefore, we should further understand the human immune system, make full use of modern advanced science and technology, and deeply explore the combined treatment strategy of PDT and immunotherapy.

Improving safety assessment remains a challenge, and further research is needed to increase the efficacy of PDT immunotherapy for effective tumor treatment. Determining the relationship between PDT and immune response in clinical studies and combining it with immunotherapy will be the focus of future research. We hope that PDT immunotherapy will prove to be an excellent cancer treatment in clinical trials.

## Author Contributions

YL contributed to the conception of the study. LT and XS performed the original draft. YL and ZH contributed significantly to review manuscript. All authors contributed to the article and approved the submitted version.

## Funding

This study was supported by frontier science program of Army Medical University (2019XQY20).

## Conflict of Interest

The authors declare that the research was conducted in the absence of any commercial or financial relationships that could be construed as a potential conflict of interest.

## Publisher’s Note

All claims expressed in this article are solely those of the authors and do not necessarily represent those of their affiliated organizations, or those of the publisher, the editors and the reviewers. Any product that may be evaluated in this article, or claim that may be made by its manufacturer, is not guaranteed or endorsed by the publisher.

## References

[1] Wang P Sun S Ma H Sun S Zhao D Wang S. Treating Tumors With Minimally Invasive Therapy: A Review. Mater Sci Eng C Mater Biol Appl (2020) 108:110198. doi: 10.1016/j.msec.2019.110198 31923997

[2] Zhang C Zhang J Shi G Song H Shi S Zhang X. A Light Responsive Nanoparticle-Based Delivery System Using Pheophorbide A Graft Polyethylenimine for Dendritic Cell-Based Cancer Immunotherapy. Mol Pharm (2017) 14(5):1760–70. doi: 10.1021/acs.molpharmaceut.7b00015 28296410

[3] Nouri Rouzbahani F Shirkhoda M Memari F Dana H Mahmoodi Chalbatani G Mahmoodzadeh H. Immunotherapy a New Hope for Cancer Treatment: A Review. Pak J Biol Sci (2018) 21(3):135–50. doi: 10.3923/pjbs.2018.135.150 30187723

[4] Huang Z. A Review of Progress in Clinical Photodynamic Therapy. Technol Cancer Res Treat (2005) 4(3):283–93. doi: 10.1177/153303460500400308 PMC1317568 15896084

[5] Biel MA. Photodynamic Therapy in Head and Neck Cancer. Curr Oncol Rep (2002) 4(1):87–96. doi: 10.1007/s11912-002-0053-8 11734119

[6] van Straten D Mashayekhi V de Bruijn HS Oliveira S Robinson DJ. Oncologic Photodynamic Therapy: Basic Principles, Current Clinical Status and Future Directions. Cancers (Basel) (2017) 9(2). doi: 10.3390/cancers9020019 PMC5332942 28218708

[7] Kamkaew A Chen F Zhan Y Majewski RL Cai W. Scintillating Nanoparticles as Energy Mediators for Enhanced Photodynamic Therapy. ACS Nano (2016) 10(4):3918–35. doi: 10.1021/acsnano.6b01401 PMC4846476 27043181

[8] Shiao SL Ganesan AP Rugo HS Coussens LM. Immune Microenvironments in Solid Tumors: New Targets for Therapy. Genes Dev (2011) 25(24):2559–72. doi: 10.1101/gad.169029.111 PMC3248678 22190457

[9] Beltran Hernandez I Yu Y Ossendorp F Korbelik M Oliveira S. Preclinical and Clinical Evidence of Immune Responses Triggered in Oncologic Photodynamic Therapy: Clinical Recommendations. J Clin Med (2020) 9(2). doi: 10.3390/jcm9020333 PMC7074240 31991650

[10] Felsher DW. Cancer Revoked: Oncogenes as Therapeutic Targets. Nat Rev Cancer (2003) 3(5):375–80. doi: 10.1038/nrc1070 12724735

[11] Oleinick NL Morris RL Belichenko I. The Role of Apoptosis in Response to Photodynamic Therapy: What, Where, Why, and How. Photochem Photobiol Sci (2002) 1(1):1–21. doi: 10.1039/b108586g 12659143

[12] Igney FH Krammer PH. Death and Anti-Death: Tumour Resistance to Apoptosis. Nat Rev Cancer (2002) 2(4):277–88. doi: 10.1038/nrc776 12001989

[13] Wu S Xing D. Mechanism of Mitochondrial Membrane Permeabilization During Apoptosis Under Photofrin-Mediated Photodynamic Therapy. J Xray Sci Technol (2012) 20(3):363–72. doi: 10.3233/XST-2012-0344 22948357

[14] Castano AP Mroz P Hamblin MR. Photodynamic Therapy and Anti-Tumour Immunity. Nat Rev Cancer (2006) 6(7):535–45. doi: 10.1038/nrc1894 PMC2933780 16794636

[15] Hayashi K Suzuki Y Fujimoto C Kanzaki S. Molecular Mechanisms and Biological Functions of Autophagy for Genetics of Hearing Impairment. Genes (Basel) (2020) 11(11). doi: 10.3390/genes11111331 PMC7697636 33187328

[16] Alzeibak R Mishchenko TA Shilyagina NY Balalaeva IV Vedunova MV Krysko DV. Targeting Immunogenic Cancer Cell Death by Photodynamic Therapy: Past, Present and Future. J Immunother Cancer (2021) 9(1). doi: 10.1136/jitc-2020-001926 PMC7802670 33431631

[17] Hanahan D Weinberg RA. The Hallmarks of Cancer. Cell (2000) 100(1):57–70. doi: 10.1016/S0092-8674(00)81683-9 10647931

[18] Krammer B. Vascular Effects of Photodynamic Therapy. Anticancer Res (2001) 21(6B):4271–7. 11908681

[19] Korbelik M Sun J Cecic I Serrano K. Adjuvant Treatment for Complement Activation Increases the Effectiveness of Photodynamic Therapy of Solid Tumors. Photochem Photobiol Sci (2004) 3(8):812–6. doi: 10.1039/b315663j 15295639

[20] Mroz P Hashmi JT Huang YY Lange N Hamblin MR. Stimulation of Anti-Tumor Immunity by Photodynamic Therapy. Expert Rev Clin Immunol (2011) 7(1):75–91. doi: 10.1586/eci.10.81 21162652 PMC3060712

[21] Kousis PC Henderson BW Maier PG Gollnick SO. Photodynamic Therapy Enhancement of Antitumor Immunity Is Regulated by Neutrophils. Cancer Res (2007) 67(21):10501–10. doi: 10.1158/0008-5472.CAN-07-1778 PMC2919236 17974994

[22] de Vree WJ Essers MC de Bruijn HS Star WM Koster JF Sluiter W. Evidence for an Important Role of Neutrophils in the Efficacy of Photodynamic Therapy *In Vivo* . Cancer Res (1996) 56(13):2908–11. 8674038

[23] Korbelik M Cecic I. Complement Activation Cascade and its Regulation: Relevance for the Response of Solid Tumors to Photodynamic Therapy. J Photochem Photobiol B (2008) 93(1):53–9. doi: 10.1016/j.jphotobiol.2008.04.005 18715798

[24] Li F Cheng Y Lu J Hu R Wan Q Feng H. Photodynamic Therapy Boosts Anti-Glioma Immunity in Mice: A Dependence on the Activities of T Cells and Complement C3. J Cell Biochem (2011) 112(10):3035–43. doi: 10.1002/jcb.23228 21678475

[25] Wachowska M Muchowicz A Demkow U. Immunological Aspects of Antitumor Photodynamic Therapy Outcome. Cent Eur J Immunol (2015) 40(4):481–5. doi: 10.5114/ceji.2015.56974 PMC4737746 26862314

[26] Preise D Oren R Glinert I Kalchenko V Jung S Scherz A. Systemic Antitumor Protection by Vascular-Targeted Photodynamic Therapy Involves Cellular and Humoral Immunity. Cancer Immunol Immunother (2009) 58(1):71–84. doi: 10.1007/s00262-008-0527-0 18488222 PMC11030999

[27] Reiter I Schwamberger G Krammer B. Activation of Macrophage Tumoricidal Activity by Photodynamic Treatment *In Vitro*–Indirect Activation of Macrophages by Photodynamically Killed Tumor Cells. J Photochem Photobiol B (1999) 50(2-3):99–107. doi: 10.1016/S1011-1344(99)00078-0 10515075

[28] Chan CW Housseau F. The ’Kiss of Death’ by Dendritic Cells to Cancer Cells. Cell Death Differ (2008) 15(1):58–69. doi: 10.1038/sj.cdd.4402235 17948029

[29] Wells AD Malkovsky M. Heat Shock Proteins, Tumor Immunogenicity and Antigen Presentation: An Integrated View. Immunol Today (2000) 21(3):129–32. doi: 10.1016/S0167-5699(99)01558-3 10689300

[30] Belicha-Villanueva A Riddell J Bangia N Gollnick SO. The Effect of Photodynamic Therapy on Tumor Cell Expression of Major Histocompatibility Complex (MHC) Class I and MHC Class I-Related Molecules. Lasers Surg Med (2012) 44(1):60–8. doi: 10.1002/lsm.21160 PMC3667415 22246985

[31] Jalili A Makowski M Switaj T Nowis D Wilczynski GM Wilczek E. Effective Photoimmunotherapy of Murine Colon Carcinoma Induced by the Combination of Photodynamic Therapy and Dendritic Cells. Clin Cancer Res (2004) 10(13):4498–508. doi: 10.1158/1078-0432.CCR-04-0367 15240542

[32] Zheng Y Yin G Le V Zhang A Chen S Liang X. Photodynamic-Therapy Activates Immune Response by Disrupting Immunity Homeostasis of Tumor Cells, Which Generates Vaccine for Cancer Therapy. Int J Biol Sci (2016) 12(1):120–32. doi: 10.7150/ijbs.12852 PMC4679404 26722223

[33] Garg AD Nowis D Golab J Vandenabeele P Krysko DV Agostinis P. Immunogenic Cell Death, DAMPs and Anticancer Therapeutics: An Emerging Amalgamation. Biochim Biophys Acta (2010) 1805(1):53–71. doi: 10.1016/j.bbcan.2009.08.003 19720113

[34] Radogna F Diederich M. Stress-Induced Cellular Responses in Immunogenic Cell Death: Implications for Cancer Immunotherapy. Biochem Pharmacol (2018) 153:12–23. doi: 10.1016/j.bcp.2018.02.006 29438676

[35] Korbelik M Sun J Cecic I. Photodynamic Therapy-Induced Cell Surface Expression and Release of Heat Shock Proteins: Relevance for Tumor Response. Cancer Res (2005) 65(3):1018–26. 15705903

[36] Korbelik M Banath J Saw KM Zhang W Ciplys E. Calreticulin as Cancer Treatment Adjuvant: Combination With Photodynamic Therapy and Photodynamic Therapy-Generated Vaccines. Front Oncol (2015) 5:15. doi: 10.3389/fonc.2015.00015 25692097 PMC4315177

[37] Butt AQ Mills KH. Immunosuppressive Networks and Checkpoints Controlling Antitumor Immunity and Their Blockade in the Development of Cancer Immunotherapeutics and Vaccines. Oncogene (2014) 33(38):4623–31. doi: 10.1038/onc.2013.432 24141774

[38] Huang L Li Y Du Y Zhang Y Wang X Ding Y. Mild Photothermal Therapy Potentiates Anti-PD-L1 Treatment for Immunologically Cold Tumors *via* an All-in-One and All-in-Control Strategy. Nat Commun (2019) 10(1):4871. doi: 10.1038/s41467-019-12771-9 31653838 PMC6814770

[39] Hu Z. The Future of Immune Checkpoint Blockade Immunotherapy: Towards Personalized Therapy or Towards Combination Therapy. J Thorac Dis (2017) 9(11):4226–9. doi: 10.21037/jtd.2017.10.31 PMC5721014 29268478

[40] Meng Z Zhou X Xu J Han X Dong Z Wang H. Light-Triggered In Situ Gelation to Enable Robust Photodynamic-Immunotherapy by Repeated Stimulations. Adv Mater (2019) 31(24):e1900927. doi: 10.1002/adma.201900927 31012164

[41] Zhang N Song J Liu Y Liu M Zhang L Sheng D. Photothermal Therapy Mediated by Phase-Transformation Nanoparticles Facilitates Delivery of Anti-PD1 Antibody and Synergizes With Antitumor Immunotherapy for Melanoma. J Control Release (2019) 306:15–28. doi: 10.1016/j.jconrel.2019.05.036 31132380

[42] Duan X Chan C Guo N Han W Weichselbaum RR Lin W. Photodynamic Therapy Mediated by Nontoxic Core-Shell Nanoparticles Synergizes With Immune Checkpoint Blockade To Elicit Antitumor Immunity and Antimetastatic Effect on Breast Cancer. J Am Chem Soc (2016) 138(51):16686–95. doi: 10.1021/jacs.6b09538 PMC5667903 27976881

[43] Gao L Zhang C Gao D Liu H Yu X Lai J. Enhanced Anti-Tumor Efficacy Through a Combination of Integrin Alphavbeta6-Targeted Photodynamic Therapy and Immune Checkpoint Inhibition. Theranostics (2016) 6(5):627–37. doi: 10.7150/thno.14792 PMC4805658 27022411

[44] Pfaar O Cazan D Klimek L Larenas-Linnemann D Calderon MA. Adjuvants for Immunotherapy. Curr Opin Allergy Clin Immunol (2012) 12(6):648–57. doi: 10.1097/ACI.0b013e32835a11d6 23090384

[45] Li L Yang S Song L Zeng Y He T Wang N. An Endogenous Vaccine Based on Fluorophores and Multivalent Immunoadjuvants Regulates Tumor Micro-Environment for Synergistic Photothermal and Immunotherapy. Theranostics (2018) 8(3):860–73. doi: 10.7150/thno.19826 PMC5771099 29344312

[46] Shirota Y Shirota H Klinman DM. Intratumoral Injection of CpG Oligonucleotides Induces the Differentiation and Reduces the Immunosuppressive Activity of Myeloid-Derived Suppressor Cells. J Immunol (2012) 188(4):1592–9. doi: 10.4049/jimmunol.1101304 PMC3273593 22231700

[47] Ni K Luo T Lan G Culbert A Song Y Wu T. A Nanoscale Metal-Organic Framework to Mediate Photodynamic Therapy and Deliver CpG Oligodeoxynucleotides to Enhance Antigen Presentation and Cancer Immunotherapy. Angew Chem Int Ed Engl (2020) 59(3):1108–12. doi: 10.1002/anie.201911429 PMC8253508 31642163

[48] Mi Y Hagan CTt Vincent BG Wang AZ. Emerging Nano-/Microapproaches for Cancer Immunotherapy. Adv Sci (Weinh) (2019) 6(6):1801847. doi: 10.1002/advs.201801847 30937265 PMC6425500

[49] Chen PM Pan WY Wu CY Yeh CY Korupalli C Luo PK. Modulation of Tumor Microenvironment Using a TLR-7/8 Agonist-Loaded Nanoparticle System That Exerts Low-Temperature Hyperthermia and Immunotherapy for in Situ Cancer Vaccination. Biomaterials (2020) 230:119629. doi: 10.1016/j.biomaterials.2019.119629 31767446

[50] Korbelik M. Cancer Vaccines Generated by Photodynamic Therapy. Photochem Photobiol Sci (2011) 10(5):664–9. doi: 10.1039/c0pp00343c 21258728

[51] Cheong TC Shin EP Kwon EK Choi JH Wang KK Sharma P. Functional Manipulation of Dendritic Cells by Photoswitchable Generation of Intracellular Reactive Oxygen Species. ACS Chem Biol (2015) 10(3):757–65. doi: 10.1021/cb5009124 25458073

[52] Gollnick SO Vaughan L Henderson BW. Generation of Effective Antitumor Vaccines Using Photodynamic Therapy. Cancer Res (2002) 62(6):1604–8. 11912128

[53] Hwang HS Shin H Han J Na K. Combination of Photodynamic Therapy (PDT) and Anti-Tumor Immunity in Cancer Therapy. J Pharm Investig (2018) 48(2):143–51 Epub 20181101. doi: 10.1007/s40005-017-0377-x PMC6323106 30680248

[54] Zhang Q Li L. Photodynamic Combinational Therapy in Cancer Treatment. J BUON (2018) 23(3):561–7. 30003719

[55] Jung NC Kim HJ Kang MS Lee JH Song JY Seo HG. Photodynamic Therapy-Mediated DC Immunotherapy is Highly Effective for the Inhibition of Established Solid Tumors. Cancer Lett (2012) 324(1):58–65. doi: 10.1016/j.canlet.2012.04.024 22554711

[56] Yang W Zhu G Wang S Yu G Yang Z Lin L. In Situ Dendritic Cell Vaccine for Effective Cancer Immunotherapy. ACS Nano (2019) 13(3):3083–94. doi: 10.1021/acsnano.8b08346 30835435

[57] Shixiang Y Xi S Junliang L Shanyi Z Xingke X Meiguang Z. Antitumor Efficacy of a Photodynamic Therapy-Generated Dendritic Cell Glioma Vaccine. Med Oncol (2011) 28 Suppl 1:S453–61. doi: 10.1007/s12032-010-9713-y 20960074

[58] Zhang L Wu S Qin Y Fan F Zhang Z Huang C. Targeted Codelivery of an Antigen and Dual Agonists by Hybrid Nanoparticles for Enhanced Cancer Immunotherapy. Nano Lett (2019) 19(7):4237–49. doi: 10.1021/acs.nanolett.9b00030 30868883

[59] Ni J Song J Wang B Hua H Zhu H Guo X. Dendritic Cell Vaccine for the Effective Immunotherapy of Breast Cancer. BioMed Pharmacother (2020) 126:110046. doi: 10.1016/j.biopha.2020.110046 32145586

[60] Korbelik M Banath J Zhang W Gallagher P Hode T Lam SSK. N-Dihydrogalactochitosan as Immune and Direct Antitumor Agent Amplifying the Effects of Photodynamic Therapy and Photodynamic Therapy-Generated Vaccines. Int Immunopharmacol (2019) 75:105764. doi: 10.1016/j.intimp.2019.105764 31352327

[61] Gollnick SO Brackett CM. Enhancement of Anti-Tumor Immunity by Photodynamic Therapy. Immunol Res (2010) 46(1-3):216–26. doi: 10.1007/s12026-009-8119-4 PMC2831137 19763892

